# A clinical case series evaluation of cross-linked gelatin foam to treat naturally occurring periodontal pockets and furcation defects in dogs

**DOI:** 10.3389/fvets.2026.1917727

**Published:** 2026-10-15

**Authors:** K. S. Stepaniuk, A. M. Sievers, A. A. Lalonde

**Affiliations:** Pet Dental Specialists, Vancouver, WA, United States

**Keywords:** canine, dog, furcation, gelatin, infrabony, periodontal, root planing, repair

## Abstract

This clinical case series evaluated periodontal debridement and the application of a biodegradable liquid scaffold composed of a porcine-derived crosslinked gelatin foam for the treatment of naturally occurring furcation defects and combined suprabony and infrabony defects in client-owned dogs. A total of 21 treated sites in 9 client-owned dogs were evaluated. Periodontal probing, intraoral computed radiography (CR), and cone beam computed tomography (CBCT) under general anesthesia were performed. Full-mouth periodontal treatment and closed (13 sites) or open (8 sites) root planing were performed before the application of the flowable scaffold. The mean duration between treatment and recheck was 169 days (with a range of 101–388 days and a median of 167 days). The mean decrease in periodontal pocket depth was 1.9 mm. The mean furcation stage decreased by 1.4, and the treated tooth periodontal stage was reduced by a mean of 0.8.

## Introduction

Periodontal disease (PD) is a ubiquitous condition initiated by plaque biofilm, resulting in the loss of attachment in the periodontium (which includes the gingiva, periodontal ligament, alveolar bone, and cementum). Periodontal disease in domestic canine patients is often not detected until it has progressed significantly, resulting in the need for tooth extraction. Advanced techniques that employ commercially available allografts, bone augmentation materials (alloplasts), and guided tissue regeneration (GTR) membranes may facilitate the preservation of affected teeth. However, there are only few commercial products available to augment closed or open-root planing treatments for intrabony pockets and furcation defects in dogs.

The prevalence of periodontal disease has historically been estimated at 80–85% in the canine population over 2 to 3 years of age (1–22). The buildup of plaque biofilm and the host’s inflammatory response result in clinical attachment loss (CAL). CAL presents as furcation bone defects in multi-rooted teeth, suprabony pockets, infrabony pockets, and combined periodontal pockets (23). Periodontal probing depths greater than 3 mm are considered periodontal pockets in dogs, and a furcation stage greater than 0 is considered abnormal. Periodontal treatment in dogs requires the mechanical removal of plaque biofilm and calculus to stall the progression of periodontal disease and encourage healing of the periodontal pocket. Many diseased teeth are identified late in the disease course due to a lack of client education, the limited ability to detect pathology during a conscious examination, lack of dental imaging, failed treatment recommendations, and poor owner compliance (23–26). Tissue healing of professionally cleaned periodontal defects occurs through the regrowth of the competing gingival epithelium, gingival connective tissue, alveolar bone, and cementum. Gingival tissues rapidly recolonize and can outcompete the preferred periodontal bone and ligament in furcation defects and infrabony pockets.

Infrabony pockets and furcation defects can be difficult to manage clinically to stop the progression of CAL in primary care veterinary practice. Depending on the level of attachment loss, the infrabony pockets require either open periodontal osseous additive surgery (e.g., bone augmentation, guided tissue regeneration) for periodontal regeneration or open periodontal osseous subtractive surgery (27, 28). Furcation defects often require open periodontal surgery and guided tissue regeneration (27–31). Commercially produced injectable products containing antimicrobials are available for augmented treatment of suprabony pockets, but they are not necessarily indicated for infrabony pockets and furcation defects to repair bone defects (32–34). GTR requires increased technical training in periodontal surgery techniques. Treatment of periodontal disease includes both professional periodontal treatments and daily home care to achieve optimal clinical results.

The investigated cross-linked gelatin foam has been clinically used in canine patients and published in the peer-reviewed literature for both dental and urinary indications in various structural formulations (35, 36). The transglutaminase cross-linked gelatin, derived from collagen, creates a porous foam scaffold for cellular migration and adhesion (37–40). The objective of this case series was to evaluate the clinical potential of an injectable version of this biodegradable scaffold composed of a porcine-derived cross-linked gelatin foam^1^ following periodontal debridement of naturally occurring furcation defects and combined suprabony/infrabony pockets in client-owned dogs. The case series included client-owned dogs, and all owners agreed to the treatment, which was standard of care, with the addition of the scaffold.

## Materials and methods

The clinical case series was performed at a single clinical veterinary specialty dentistry and oral surgery practice. The objective was to recruit 10 client-owned dogs with a minimum of one furcation defect or combined suprabony/infrabony pocket of a strategic tooth [maxillary canine teeth (104, 204), mandibular canine teeth (304, 404), maxillary fourth premolars (108, 208), mandibular first molars (309, 409)]. Inclusion criteria included patients for whom owner consent was obtained for use of the product following standard root planing and whose owners had agreed to a post-treatment clinical conscious oral examination recheck 2 weeks following treatment and to a post-treatment reevaluation under general anesthesia with imaging. Cone-beam computed tomography (CBCT) images were obtained with a Planmeca Verity Vet^2^ system using Planmeca Romexis viewing software (version 6.3.0.2015; SMQS, Glattburg, Switzerland), ^3^ and computed intraoral digital radiographs (CR)^4^ were obtained, and periodontal probing was completed during initial diagnosis and treatment, as well as reassessment. The acquired images were viewed on a dedicated high-resolution monitor^5^ in an appropriately lit room. When appropriate, the images were assessed at the highest resolution of an unstitched scan (voxel resolution of 200 μm and 16 cm × 16 cm field of view). If necessary for larger patients, images were assessed using a digitally stitched scan with a voxel resolution of 400 μm and up to a 21.2 cm × 21.2 cm field of view. A complete dental cleaning and periodontal debridement were necessary at the time of treatment. Patients were required to be medically stable prior to general anesthesia and have no co-morbidities and/or medications that would interfere with the required general anesthesia, periodontal surgery, and/or periodontal surgical wound healing.

All patients had complete physical examinations and preoperative laboratory evaluation for general anesthesia. All patients received multimodal anesthesia/analgesia, regional analgesia with 0.5% bupivacaine/1:200000 epinephrine^6^, thermoregulation, and a dedicated anesthetist for anesthetic monitoring and interventions. Each patient received radiographic imaging assessment. Periodontal probing, furcation, and staging measurements were documented using American Veterinary Dental College (AVDC®) criteria with a surgical loupe-magnified examination and a UNC-15/23 dental periodontal probe (Boxes 1, 2) (41). The periodontal stage was assigned to each treated tooth based on the most severe periodontal probing depth and bone loss percentage based on imaging. Using surgical loupes and lights, the periodontal defects were cleaned with an ultrasonic scaler and periodontal curettes to remove all visible inflammatory tissue, subgingival plaque, subgingival calculus, and diseased cementum (if necessary) from the root surfaces and juxtaposed alveolar bone with closed root planing (13 sites) and open root planing (8 sites). Open root planing was performed after creation and elevation of an envelope flap using a #15 scalpel blade and EX9 periosteal elevator. Eleven, nine, and one treatment sites were treated by Alice M. Sievers (AS), Kevin S. Stepaniuk (KS), and Angele A. Lalonde (AL), respectively.

Box 1 Periodontal staging criteria (41). Reproduced with permission of American Veterinary Dental College^®^.

**Table:** 

Periodontal stages	Severity of periodontal disease (PD) relates to the treated tooth
Normal (PD0)	Clinically normal; gingival inflammation or periodontitis is not clearly evident.
Stage 1 (PD1)	Gingivitis only without attachment loss; the height and architecture of the alveolar margin are normal.
Stage 2 (PD2)	Early periodontitis: less than 25% attachment loss or, at most, there is a stage 1 furcation involvement of multirooted teeth. There are early radiological signs of periodontitis. The loss of periodontal attachment is less than 25% as measured either by probing of the clinical attachment level, or by radiographic (imaging) determination of the distance of the alveolar margin from the cementoenamel junction relative to the length of the root.
Stage 3 (PD3)	Moderate periodontitis: 25–50% of attachment loss as measured either by probing of the clinical attachment level, or by radiographic (imaging) determination of the distance of the alveolar margin from the cementoenamel junction relative to the length of the root, or there is stage 2 furcation involvement of multirooted teeth.
Stage 4 (PD4)	Advanced periodontitis: >50% attachment loss as measured either by probing of the clinical attachment level, or by radiographic (imaging) determination of the distance of the alveolar margin from the cementoenamel junction relative to the length of the root, or there is stage 3 furcation involvement of multirooted teeth.

Box 2 Periodontal probing furcation indices (41). Reproduced with permission of American Veterinary Dental College^®^.

**Table:** 

Furcation index	Description for furcation classification
Stage 1 (F1)	Periodontal probe extends less than halfway under the crown in any direction of a multirooted tooth with attachment loss.
Stage 2 (F2)	Periodontal probe extends greater than halfway under the crown in any direction of a multirooted tooth with attachment loss but not through and through.
Stage 3 (F3)	Periodontal probe extends under the crown of a multirooted tooth, through and through from one side of the furcation to the other.

Sterile isotonic saline was used for surgical irrigation. The gelatin scaffold was provided as an 800 mg dry sterile powder in a syringe to be mixed with saline, at the point of care, to generate a flowable foam per the manufacturer’s instructions. Following periodontal debridement and root planing, the foam was injected into the periodontal defect with a 20-gauge disposable pre-bent dental applicatory tip.^7^ Gentle pressure was applied to the gingival tissue to adapt the gingival tissues to the tooth surface over the scaffold after application. All open root-planed sites were sutured with 4–0 polyglecaprone 25^8^ to adapt the gingival tissue to the treated tooth surface following application of the foam. Five of the 13 closed root-planed sites had a 5–0 polyglecaprone 25^9^ periodontal suture placed to approximate and to support gingival tissue following digital pressure to re-adapt the gingiva to the treated tooth surface.

A subcutaneous post-operative injection of either 0.1 mg/kg of meloxicam^10^ or 2.2 mg/kg of carprofen^11^ was administered during anesthetic recovery. All patients were discharged with analgesia. Either the non-steroidal anti-inflammatory drug (NSAID) meloxicam^12^ or carprofen^13^ was prescribed for 3–5 days at 0.1 mg/kg every 24 h or 2.2 mg/kg every 12 h per os, respectively. One patient received grapiprant^14^ 60 mg orally postoperatively since they were already receiving that medication for osteoarthritis. One patient did not receive an NSAID due to unrelated medical reasons. The patients were prescribed gabapentin^15^ (10 mg/kg per os every 8–12 h for 7 days) as needed for additional pain control. Nine of the ten patients were prescribed doxycycline^16^ 4–5 mg/kg per os every 12–24 h for 5–7 days postoperatively. A 0.13% chlorhexidine gluconate oral rinse^17^ every 12 h for 10 days post-operatively was prescribed to start 48 h after surgery in seven patients. It was recommended that the patient’s regular diet be changed to a soft formulation (canned equivalent food or moistened kibble) for 7–10 days post-operatively. Home care was recommended to be delayed for 10–14 days to allow for post-surgical healing. The patients were scheduled for a conscious oral examination recheck of the surgical site 2–3 weeks following surgery. An anesthetized recheck for diagnostic imaging and periodontal probing was recommended to be scheduled in 4 months. A two-tailed *paired-samples t*-test for means^18^ was used to evaluate the furcation and periodontal pockets for treatment changes in this case series.

Rescue treatments were planned in the event of complications or treatment failures. In the event the periodontal procedure(s) were unsuccessful, defined as decreased attachment height (progressing periodontal disease), surgical extraction or additional periodontal surgery with bone augmentation or guided tissue regeneration was to be offered.

## Results

There were no product exposures, evidence of infection at the treatment site, or complications observed at the scheduled 2–3-week recheck. All periodontal surgery sites were healed, and daily home care was recommended. A review of medical records found that 56% of the patients had received some form of home care prior to the recheck evaluation (e.g., tooth-brushing, dental chews, water additives).

Treatment occurred in ten client-owned dogs. One patient was excluded from the study because the patient failed to return for the scheduled anesthetized recheck. During telephone communication, the client reported the dog had developed unrelated medical issues that they wanted managed prior to another procedure. Nine dogs with a mean age of 8.1 years (range 4.2–13.5 years; median 8.2 years) were included in the evaluation. There were five male-altered and four female-altered patients. The mean patient weight was 12.9 kg (range 5.0–28.3 kg, median 7.1 kg). Therefore, nine patients and 21 treated sites (10 furcation sites, 11 periodontal pockets) were included in the data analysis. The average time between treatment and anesthetized recheck was 169 days (range 101–388 days, median 167 days).

The mean pre-operative canine tooth periodontal probing depth was 5.5 mm with a statistically significant (*p* < 0.05) attachment gain of 1.9 mm (Table 1) at recheck. One site (tooth 204) had an increased palatal pocket depth from 7 mm to 8 + mm, resulting in an oronasal fistula. Tooth 204 was surgically extracted with closure of the oronasal fistula. The mean pre-treatment furcation index was 2.1, and the mean furcation index at reevaluation was 0.7, representing a statistically significant (*p* < 0.05) improvement (Table 2). Of the 21 treated teeth, more than half (57%, *p* < 0.05) showed improvement in periodontal disease stage, and 38% were stable (Figure 1 and Table 3). Subjective new bone density was observed at 16 treated teeth (Figures 2, 3).

**Table 1:** Periodontal probing depths of periodontal pockets in the treated canine teeth [mesial (M), mesial palatal (MP), palatal (P), lingual (L), mesial lingual (ML), distal lingual (DL)].

Treated site	Tooth number	Pocket depth (mm) at treatment	Pocket depth (mm) at recheck	Change in pocket depth mm at recheck	Pocket location
4	104	5	3	−2	P
5	204	4	2	−2	M
7	104	5	3	−2	MP
8	204	4	3	−1	MP
9	304	5	5	0	ML
10	404	4	3	−1	ML
14	104	5	4	−1	P
15	204	7	8	1	L, DL
16	104	9	3	−6	ML
17	204	7	3	−4	ML
19	204	5	2	−3	M, MP

**Table 2:** Furcation stages of treated multirooted strategic teeth according to the American veterinary dental college (AVDC®) [Buccal (B), Palatal (P), Lingual (L)].

Treated site	Tooth number	AVDC® furcation stage at treatment	AVDC® furcation stage at recheck	AVDC® change in furcation stage at recheck	Furcation location
1	208	2	1	−1	P
2	108	2	0	−2	P
3	208	2	0	−2	P
6	208	3	1	−2	B
11	108	2	1	−1	P
12	309	2	1	−1	L
13	409	2	2	0	L
18	208	2	0	−2	L
20	208	1	1	0	B
21	208	3	0	−3	B

**Figure 1:**
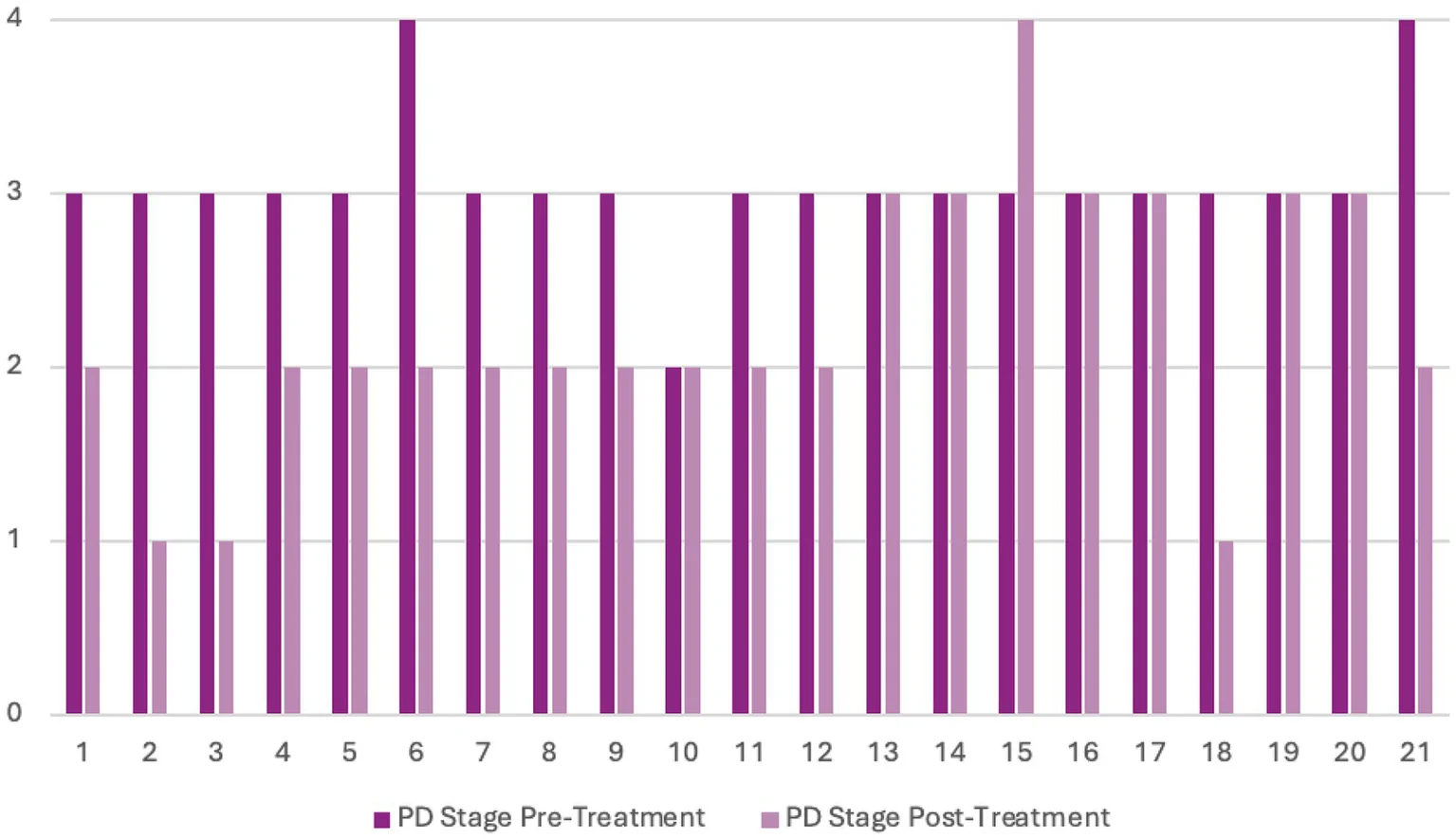
Individual tooth periodontal stages measured with periodontal probing and imaging of the 21 treated teeth.

**Table 3:** Periodontal disease (PD) stage summary of 21 treated sites with root planing closed (RP/C) or root planing open (RP/O) prior to gelatin foam treatment.

Treatment site	Age (years)	Breed	Tooth number	PD stage pre-treatment	PD stage post-treatment	Root planing
1	8.7	Cavalier King Charles mix	208	3	2	RP/O
2	8.2	Mix	108	3	1	RP/O
3	8.2	Mix	208	3	1	RP/O
4	4.2	Australian cattle dog mix	104	3	2	RP/O
5	4.2	Australian Cattle Dog Mix	204	3	2	RP/O
6	4.2	Australian cattle dog mix	208	4	2	RP/O
7	5.8	Italian greyhound	104	3	2	RP/C
8	5.8	Italian Greyhound	204	3	2	RP/C
9	5.8	Italian greyhound	304	3	2	RP/C
10	5.8	Italian greyhound	404	2	2	RP/C
11	5.8	Italian greyhound	108	3	2	RP/C
12	5.8	Italian greyhound	309	3	2	RP/C
13	5.8	Italian greyhound	409	3	3	RP/C
14	6	Italian Greyhound	104	3	3	RP/C
15	6	Italian greyhound	204	3	4	RP/C
16	9.5	Australian cattle dog	104	3	3	RP/O
17	9.5	Australian cattle dog	204	3	3	RP/O
18	8.2	Labrador retriever	208	3	1	RP/C
19	8.7	Pomeranian	204	3	3	RP/C
20	8.7	Pomeranian	208	3	3	RP/C
21	13.5	Australian shepherd	208	4	2	RP/C

**Figure 2:**
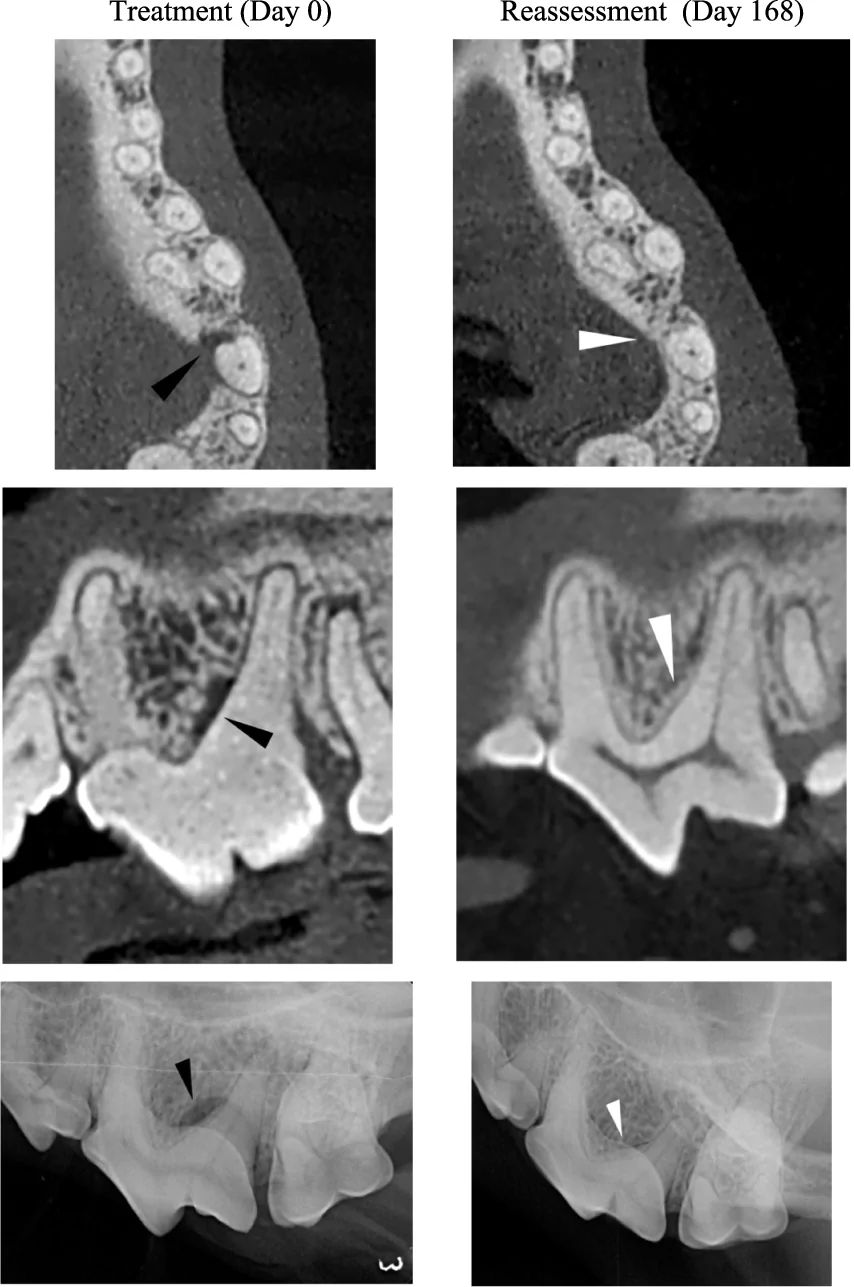
Representative cone-beam computed tomography (CBCT) images and computed radiography (CR) intraoral radiographs of site #3 treated with open root planing (RP/O), the left maxillary fourth premolar (208). Note the periodontal bone defect (black arrowheads) on the treatment day and the subjective increased bone density at reassessment (white arrowheads).

**Figure 3:**
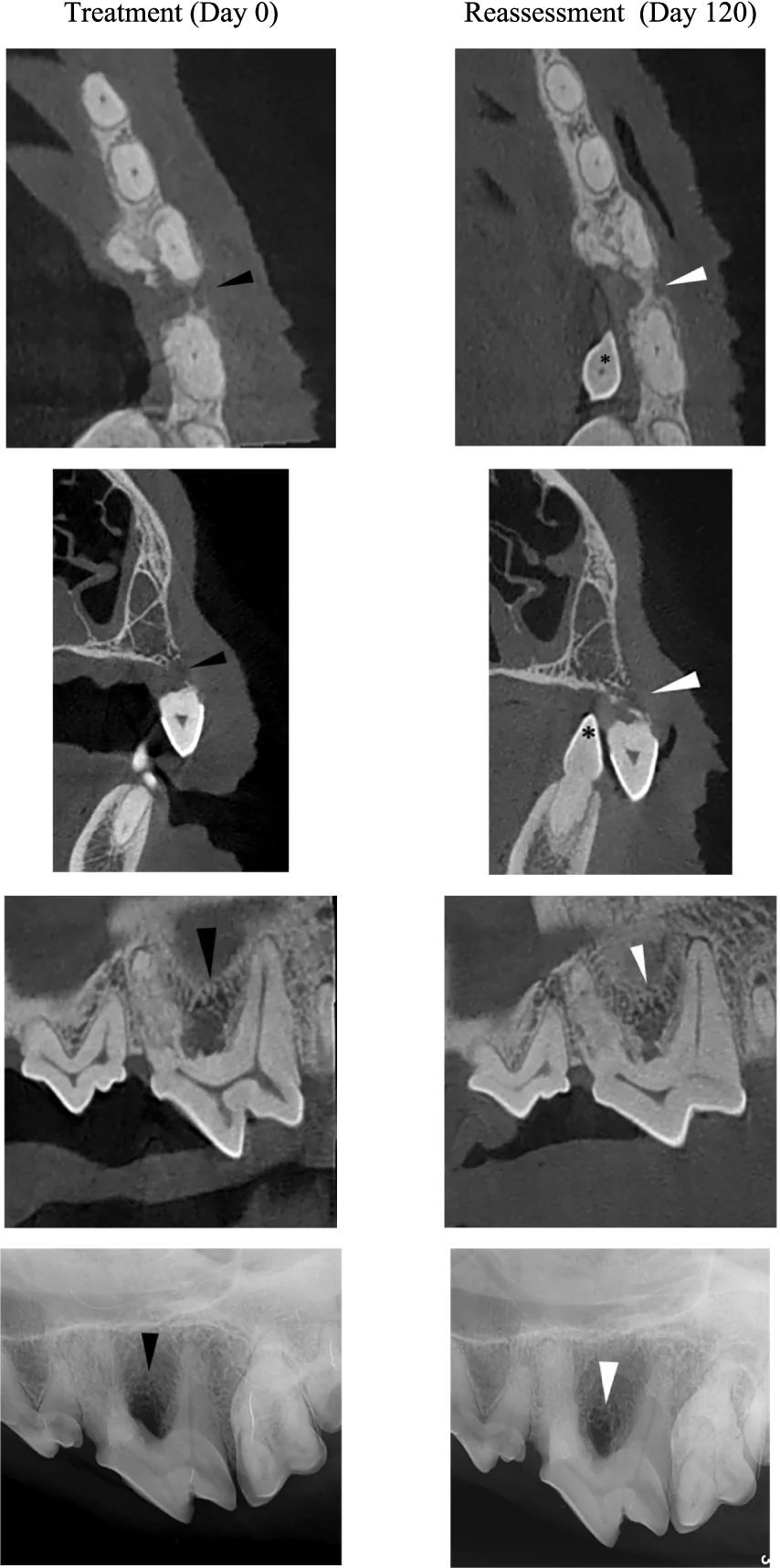
Representative cone-beam computed tomography (CBCT) images and computed radiography (CR) intraoral radiographs of site #21 treated with closed root planing (RP/C), the left maxillary fourth premolar (208). Note the periodontal bone defect (black arrowheads) on the treatment day and the subjectively increased bone density at reassessment (white arrowheads). Due to the patient having a reduced gape angle of the temporomandibular joint in the reassessment image, the mesial cusp of the left mandibular first molar (309) is visible in the post-treatment images (black asterisk).

## Discussion

The periodontal tissues include gingiva, periodontal ligament, cementum, and alveolar bone. Periodontal tissue clinical attachment loss results from the subgingival plaque biofilm and the associated host inflammatory response. CAL of the periodontal tissues results in furcation involvement and exposures, infrabony pockets, suprabony pockets, and/or combined periodontal pockets. Treatments may include periodontal cleanings, closed root planing, open root planing, bone augmentation, platelet-rich plasma, guided tissue regeneration, and an assortment of preclinical emerging techniques and products (27–31). Gingival epithelium, gingival connective tissue, alveolar bone, and periodontal ligament compete during healing of periodontal bone and/or furcation defects. The most desirable tissues for re-attachment are new periodontal ligament and bone. However, junctional epithelium, gingival connective tissue, and gingival epithelium can re-colonize the defect faster, preventing the preferred osteoblasts, fibroblasts, and undifferentiated mesenchymal cells from entering the treated site and thereby creating a more organized and anatomically appropriate tissue repair.

Many veterinary patients suffer from hidden, untreated periodontal pockets and furcation defects. Patients are often presented for dental cleaning late in the course of progressive periodontal disease, limiting treatment options and typically resulting in extractions. Periodontal debridement and closed or open root planing with or without adjunctive treatments may be used to manage periodontal disease. There are limited controlled studies and few published case series evaluating techniques and materials to stabilize and/or improve attachment loss of periodontal defects in veterinary patients. Many clients are interested in preserving strategic teeth, and treatment options to improve or stabilize clinical attachment levels should be considered when appropriate. Patient welfare should be a priority in decision-making for the treatment of strategic teeth. Zacher et al. (42) summarized favorable and unfavorable characteristics for maintaining teeth with furcation lesions. Client education and confirmation of commitment for appropriate future home care are critical for the best long-term outcomes; this discussion should occur prior to performing advanced periodontal treatments to save teeth. Future anesthetized periodontal treatment and ongoing maintenance are necessary throughout all life stages of veterinary patients to maintain periodontal attachment and prevent future CAL.

All periodontal treatments begin with a professional periodontal cleaning with attention to removal of subgingival plaque, calculus, and inflammatory tissue. Complete removal of inflammatory tissue and the subgingival deposits, while preserving dentin, is necessary for treatment. The goal of treatment is to stabilize the CAL and/or increase attachment gain, thereby diminishing periodontal pocket depth to improve home care success and professional maintenance. Treatments that decrease pocket depth and improve attachment gain could result in different histologic outcomes. Tissues may be repaired by competing periodontal tissues in a disorganized architecture, or true periodontal regeneration may occur, with regeneration of natural tissue structures consistent with native architecture (43). Regardless of whether the end result is repair or regeneration, stabilizing or eliminating clinical periodontal defects with new attachment can be considered a clinical success. Re-entry into clinical patient treatment sites to assess the type(s) of tissue(s) that have diminished the periodontal pocket is not routinely performed because removing the tooth and supporting bone/tissue for assessment defeats the purpose of the clinical treatment. Therefore, assessment of success is based on stable or improved periodontal measurements and stable or improved bone density assessed by imaging techniques. Regeneration of native tissue may be the ultimate goal of periodontal treatment; however, repair with various periodontal tissues of the defect may occur instead.

Periodontal debridement treatment for the periodontal pockets and furcation defects in this case series occurred prior to the adjunctive gelatin foam placement. Open mucoperiosteal periodontal surgery should be considered for deep periodontal pockets greater than 5 mm to improve surgical visualization and facilitate complete cleaning of the root surface. Similarly, furcation defects may be difficult to completely clean, and to remove all plaque and calculus deposits from the avascular root surface, so open mucoperiosteal periodontal surgery may be necessary to assess and treat all root surfaces.

In this study, there was one clinically treated tooth failure that required surgical extraction of the tooth and closure of the inapparent oronasal fistula defect during the anesthetized recheck. With retrospective clinical decision evaluation, the failed maxillary canine tooth was a poor candidate for treatment. Several factors may have contributed to the lack of success for this tooth, including the depth of the defect in relation to the overall remaining palatal bone, closed root planing resulting in less than desirable periodontal subgingival scaling, difficult home care on the palatal aspect of the tooth, lack of non-steroidal anti-inflammatories (NSAIDs), and the anatomically thin maxillary palatal bone in the patient. The palatal periodontal pocket was 7 mm, extending close to the anatomical limits of the assessed normal thin palatal bone attachment in the breed (Italian Greyhound). Treatment of the contralateral canine tooth in this patient was successful. Therefore, the individual periodontal anatomical defect and clinical decision regarding case selection by the operator are the presumed reasons for failure. Although the study design allowed some flexibility in post-operative medications, the individual patient requiring extraction at recheck does demonstrate the need for more tightly controlled post-operative medication planning in future studies, as this was the only patient unable to receive a non-steroidal anti-inflammatory.

Similarly, doxycycline was prescribed post-operatively for matrix metalloproteinase inhibition in periodontal disease treatment for all but one patient (44). It is acknowledged that it is difficult to control every clinical treatment variable in client-owned patient case series compared to laboratory-controlled patient populations.

One patient failed to return for the recommended 4-month anesthetized recheck. After several communications, the anesthetized recheck and evaluation was scheduled 388 days following the initial treatment, making the average treatment recheck 169 days for the study. That individual patient’s furcation stage decreased from stage 2 to stage 1. Daily home care is recommended to help maintain clinical attachment gains after periodontal treatment. It was reported that this patient had received no home care. It is possible that this patient may have had increased initial attachment gains, after which CAL recurred as the year progressed. This patient emphasizes the importance of adherence to scheduled professional care recommendations and daily home care as part of comprehensive periodontal treatment plans. Simply treating periodontal disease once and expecting attachment gain to improve and stabilize without lifelong management is naïve. Even with new treatment options that allow preservation of teeth that might otherwise be extracted, it is important to emphasize that periodontal disease requires lifelong management, including repeated treatment under anesthesia. Many individual, mechanical, nutritional, functional, age-related, and immune-system factors influence periodontal CAL progression and responses to treatment or stabilization efforts (23).

The scaffold used in this study is a three-dimensional elastic foam composed of enzymatically cross-linked gelatin designed to fill defects and provide mechanical support for tissue repair. Gelatin, derived from collagen, exhibits lower immunogenicity and preserves cellular binding sites, allowing for migration of surrounding periodontal cells (37–39, 45, 46). The scaffold was applied directly into the root-planed furcation and intrabony defects. The flowable product undergoes *in situ* enzymatic crosslinking, creating a stabilized foam scaffold. The resulting architecture is designed to spatially organize tissue regrowth and facilitate repair of the defect with periodontal cellular tissue migration, adhesion, and proliferation. The clinical periodontal stage was successfully stabilized (38%) or improved (57%) following root planing and scaffold treatment in 95% of the treated sites.

Gawor et al. (36) published a clinical study of a novel bioabsorbable scaffold made of porcine cross-linked gelatin^19^ in a split-mouth clinical design. Decreased probing depths were noted with statistical improvement in the scaffold-treated teeth (36). Similar to that study, in this case series, the scaffold-treated teeth had improved attachment gain, as measured with periodontal probing depths, with application of flowable gelatin scaffold following periodontal root planing. In Gawor et al. (36), furcation treatment was reported to remain at the same stage at a 90-day reassessment period for both teeth treated with open periodontal therapy and teeth treated with open periodontal therapy and placement of the dry porcine gelatin scaffold. Gawor et al. (36) also stated that veterinary studies with longer follow-up times are needed. In this clinical case series of patients with a longer follow-up time and a modified liquid formulation of the scaffold product applied to the advanced defects, there was an identified trend of statistically significant improvement in stage of furcation involvement and periodontal probing depth. Both the previous clinical study and this clinical study involved relatively small populations and many treatment variables from periodontal surgery techniques, imaging modality, home care, recheck time frames, application of scaffold materials, formulation of the scaffold, and/or retention of scaffold materials that could alter treatment outcomes. Controlled laboratory or clinical studies, with larger populations, could be designed to help elucidate the variance. Further clinical studies with the scaffold to assess furcation treatments should be considered.

Scaffold retention, if necessary, may be improved with adjunctive periodontal surgery techniques. Following treatment and application of scaffolds to treated furcation defects, a coronally advanced flap (CAF), with periodontal sutures, may improve initial scaffold adaptation against the avascular cleaned root surfaces. The CAF could theoretically allow for better initial scaffold retention and potentially improve clinical attachment gain. Future studies could evaluate this hypothesis.

This porcine-derived gelatin is a xenograft (27, 28). Regardless of whether an allograft or xenograft is used, the development of the body’s natural fibrin clot after meticulous subgingival cleaning is important in periodontal wound healing. The gelatin resorbs over time and is replaced with the patient’s own tissues. The nature and architecture of the new tissue involved in the periodontal attachment is not known at this time.

Clinical studies in client-owned patients introduce many variables to control and account for when assessing data and results. Limitations in this study include, but are not limited to, no re-entry into the clinical sites to determine the histological nature of the tissues that decreased the periodontal defects, a single patient that did not receive post-operative NSAIDs, the absence of comparative computer-aided bone density imaging evaluation, the inability to use a split-mouth clinical design for individual patient controls with identical and naturally occurring contralateral matched defects, variable post-operative medications, and the inability to ensure both patient and owner compliance for similar post-operative daily home care periodontal maintenance. Regardless of potentially confounding factors, with a single exception, the clinical periodontal defects stabilized or improved as measured by clinical periodontal probing. The treatments were successful in stabilizing or decreasing naturally occurring periodontal defects, both in the periodontal pockets and furcation defects, in this small group of patients with CAL.

Periodontal disease is extremely common in canine patients (47). It is very important to understand the classification of the periodontal defect(s) to be treated, treatment options available, appropriate selection of clinical cases for treatment, and the application of materials that could augment traditional periodontal cleaning, closed root planing, and/or open root planing. Understanding the patient signalment, skull conformation, patient and owner compliance, age and stage of diagnosis of periodontal defects, the biofilm, and the host inflammatory response for multifactorial periodontal attachment loss and for treatment planning is necessary (48). The use of alloplasts, allografts, and xenografts for treatment of periodontal defects is an evolving area of practice, as well as a slow process to evaluate and measure clinical success. Cleaning furcation defects can be difficult and dependent on individual tooth morphology, the stage of furcation, and access to the furcation within the oral cavity (42, 49). Therefore, it is very important that the veterinary clinician understand the complexity of the multifactorial periodontal disease pathophysiology and the treatment options available to appropriately select clients, patients, strategic teeth, and individual treatment techniques for each individual’s periodontal defect in the veterinarian’s clinical decision-making process.

## Conclusion

Following standard periodontal debridement, a clinical application of a novel flowable scaffold, composed of porcine-derived cross-linked gelatin foam, was successful in stabilizing and/or improving periodontal attachment in naturally occurring furcation bone defects and combined suprabony and infrabony pockets. This point-of-care treatment offers a potential adjunctive benefit following skillful periodontal debridement for teeth with periodontal bone loss. While there are inherent limitations in clinical veterinary patients with naturally occurring periodontal defects, further clinical studies in dogs, with an increased number of treatment sites and, ideally, matched controls, could address many of these limitations.

## Data Availability

The raw data supporting the conclusions of this article will be made available by the authors, without undue reservation.

## Footnotes

1 Flowable cross-linked gelatin foam, BioChange™ Ltd., Yokneam, Isreal.

2 Planmed Verity® VET, Planmeca, Helsinki, Finland.

3 Romexis, Planmeca®, Helsinki, Finland.

4 iM3® CR7 Vet Digital X-Ray, iM3®, Vancouver, USA.

5 S2722QC Flat Panel Monitor, Dell, Austin, Texas, USA.

6 Marcaine™ 0.5% with 1:200000 epinephrine, Hospira, Lakeforest, IL, USA.

7 Application canulae type 41, Voco, Cuxhaven, Germany.

8 4–0 Polyglecaprone 25, Monocryl®, Ethicon, Somerville, NJ, USA.

9 5–0 Polyglecaprone 25, Monocryl®, Ethicon, Somerville, NJ, USA.

10 Meloxicam, OstiLox™, Norbrook Laboratories, Newery, Northern Ireland.

11 Carprofen, OstiFen™, Norbrook Laboratories, Newery, Northern Ireland.

12 Meloxicam oral suspension, Meloxidyl®, MWI/VetOne, Boise, ID, USA.

13 Carprofen, Rimadyl®, Zoetis, Lincoln, NE, USA.

14 Grapiprant, Galliprant®, Elanco, Greenfield, IN.

15 Gabapentin capsules, Amneal®, Amneal Pharmaceuticals LLC, Bridgewater, NJ, USA.

16 Doxycycline hyclate, Zydus Life Sciences Ltd., Ahmedabad, India.

17 Clenz-A-Dent™ chlorhexidine gluconate 0.13% oral rinse, Ceva Animal Health LLC, Lenexa, KS, USA.

18 Microsoft® Excel for MAC, Version 16.107.4, Microsoft Corporation, Redmond, WA, USA.

19 ReGum™Vet, BioChange™ Ltd., Yokneam, Isreal.
